# Abdominal applications of ultrasound fusion imaging technique: liver, kidney, and pancreas

**DOI:** 10.1186/s13244-019-0692-z

**Published:** 2019-01-28

**Authors:** European Society of Radiology (ESR), Mirko D’Onofrio, Alessandro Beleù, Diana Gaitini, Jean-Michel Corréas, Adrian Brady, Dirk Clevert

**Affiliations:** Vienna, Austria

**Keywords:** Ultrasound, Fusion imaging, Liver, Oncologic imaging, Tumor ablation

## Abstract

Fusion imaging allows exploitation of the strengths of all imaging modalities simultaneously, eliminating or minimizing the weaknesses of every single modality. Ultrasound (US) fusion imaging provides benefits in real time from both the dynamic information and spatial resolution of the normal US and the high-contrast resolution and wider field of view of the other imaging methods. US fusion imaging can also be associated with the use of different ultrasound techniques such as color Doppler US, elastography, and contrast-enhanced US (CEUS), for better localization and characterization of lesions. The present paper is focused on US fusion imaging technologies and clinical applications describing the possible use of this promising imaging technique in the liver, kidney, and pancreatic pathologies.

## Key points

- Fusion imaging helps in the detection and localization of lesions with low conspicuity on standard B-mode US.
- US fusion imaging can also be associated with the use of different ultrasound techniques such as color Doppler US, elastography, and contrast-enhanced US (CEUS).
- The current principal use of US fusion imaging is during hepatic interventional procedures. However, new applications in both intra- and extra-abdominal areas are emerging more and more.

## Introduction

Taking advantage of various imaging techniques to improve both diagnosis and interventional procedures has become a very common process and is an integral part of the work of the modern radiologist. Normally, the association process is a mental act that involves the integration of information coming from multiple imaging methods such as ultrasound (US), computed tomography (CT), magnetic resonance (MR), and positron emission tomography (PET).

During interventional procedures, this process is referred to as “cognitive fusion” or “visual registration” and consists of the careful studying of an examination acquired before the procedure, usually CT or MR, and the subsequent use of US as a guide for the performance of the procedure, after mental superimposition of the spatial information from the prior study [1]. However, this process can be difficult if the ideal US scanning plane is different to the classical orthogonal CT or MR image. Moreover, breathing and displacement and deformation of the abdominal structures due to pressure from the US probe can affect the process of mental registration [2].

Thanks to the recent improvements of technology and computing power, a real-time computerized fusion of radiological images has been developed and implemented in modern high-end US machines, to allow synchronous association of US images with one or more other cross-sectional studies such as CT, MR, or PET, which are instantly reconstructed in the corresponding plane.

US guidance is still the guidance method of choice for percutaneous interventional procedures, as it provides real-time imaging, does not use ionizing radiation, is easily accessible, and is cheap [3]. However, compared to CT and MR, it has lower contrast resolution and a narrower field of view and is affected by the presence of gas and fat. The use of real-time fusion imaging allows exploitation of the strengths of all imaging modalities simultaneously, eliminating or minimizing the weaknesses of every single modality. Therefore, US fusion imaging provides benefits in real time from both the dynamic information and spatial resolution of the normal US and the high-contrast resolution and wide field of view of the other imaging methods. US fusion imaging can also be associated with advanced US imaging techniques such as color Doppler US, elastography, and contrast-enhanced US (CEUS), for better localization and characterization of lesions to be treated [4].

## Fusion imaging technology

There are several available spatial tracking methods for US probes, including optical, image-based, and electromagnetic tracking [1]. The electromagnetic tracking system is the one mostly used for percutaneous interventional procedures. It comprises a magnetic field generator, located 20–30 cm from the patient, and a position sensor attached to the probe, or integrated into the needle. When the position sensor is moved in the magnetic field, an induced electric current is generated, allowing the system to recognize its 3D spatial position and orientation.

The image fusion procedure begins with the importation of data from a previous CT/MR/PET exam. Next is the planning phase, which consists of several steps to study the target lesions and the structures involved in the procedure and to establish the spatial orientation of the patient with respect to the probe. To do this, both anatomical landmarks and external markers can be used. Using anatomical landmarks alone, synchronization of the US images with CT/MR must be manual and requires the identification of motionless anatomical structures on the US (e.g., vessels, cysts, calcifications) that are then manually matched on the tomographic exam [5]. If external markers are used, placed on the patient’s skin during the CT acquisition phase, the image coupling process will be automatic, faster, and more reliable.

**Fig. 1:**
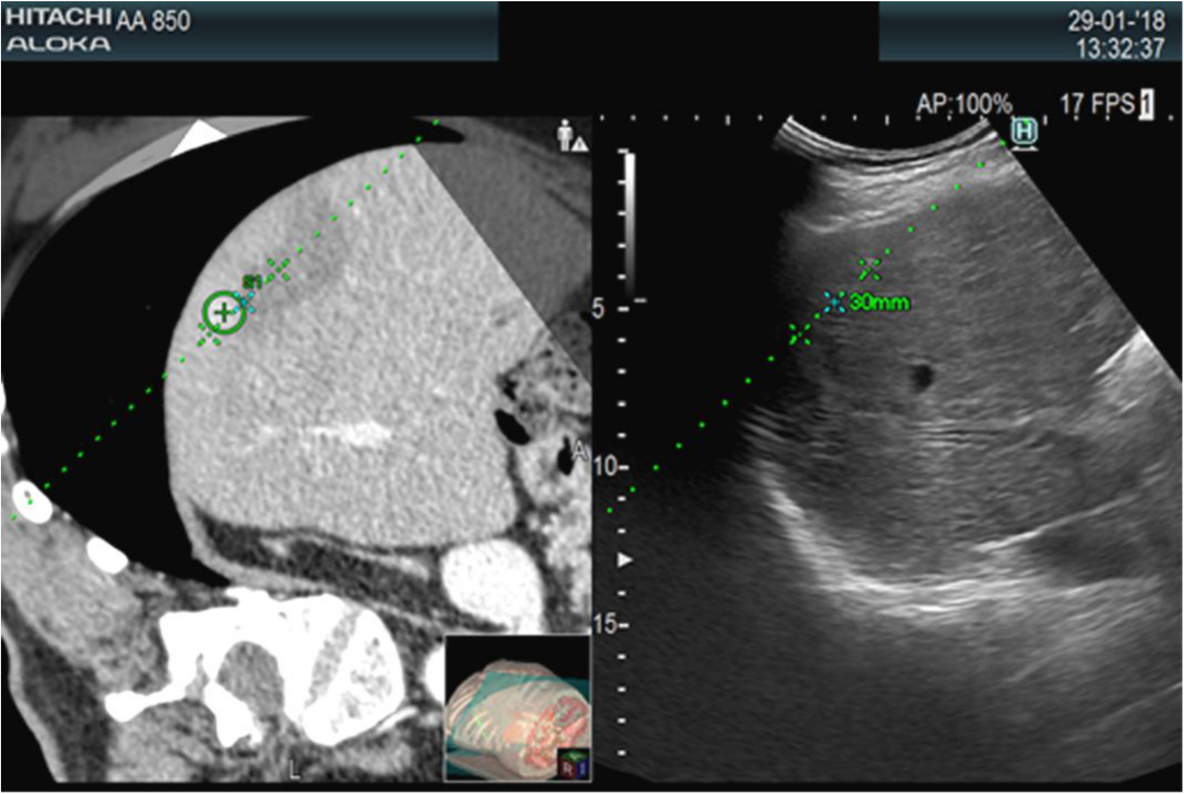
CT-US fusion imaging. Treatment of very small hypervascular nodular recurrence of HCC adjacent to a previously ablated area

**Fig. 2:**
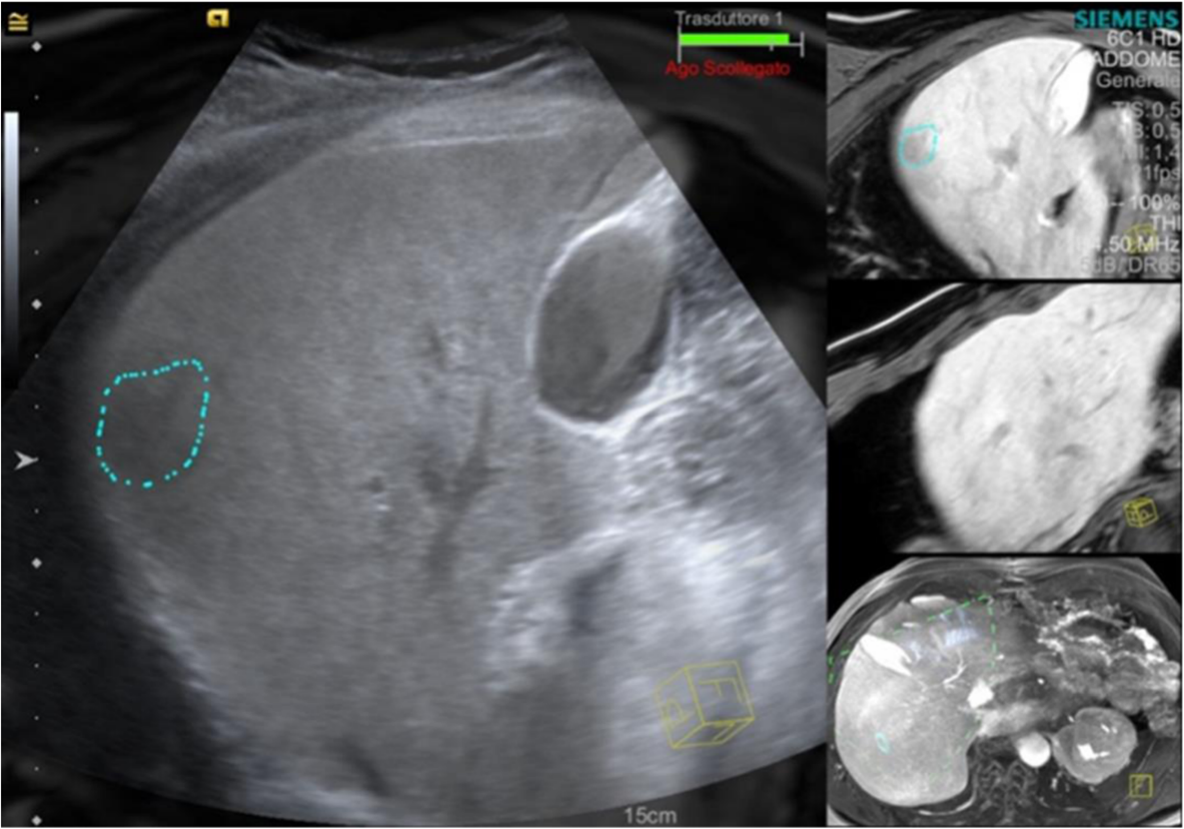
MR-US fusion imaging. Biopsy of very small liver nodule, hypointense on the late hepatobiliary phase of MR, with a final diagnosis of a metastatic lesion

**Fig. 3:**
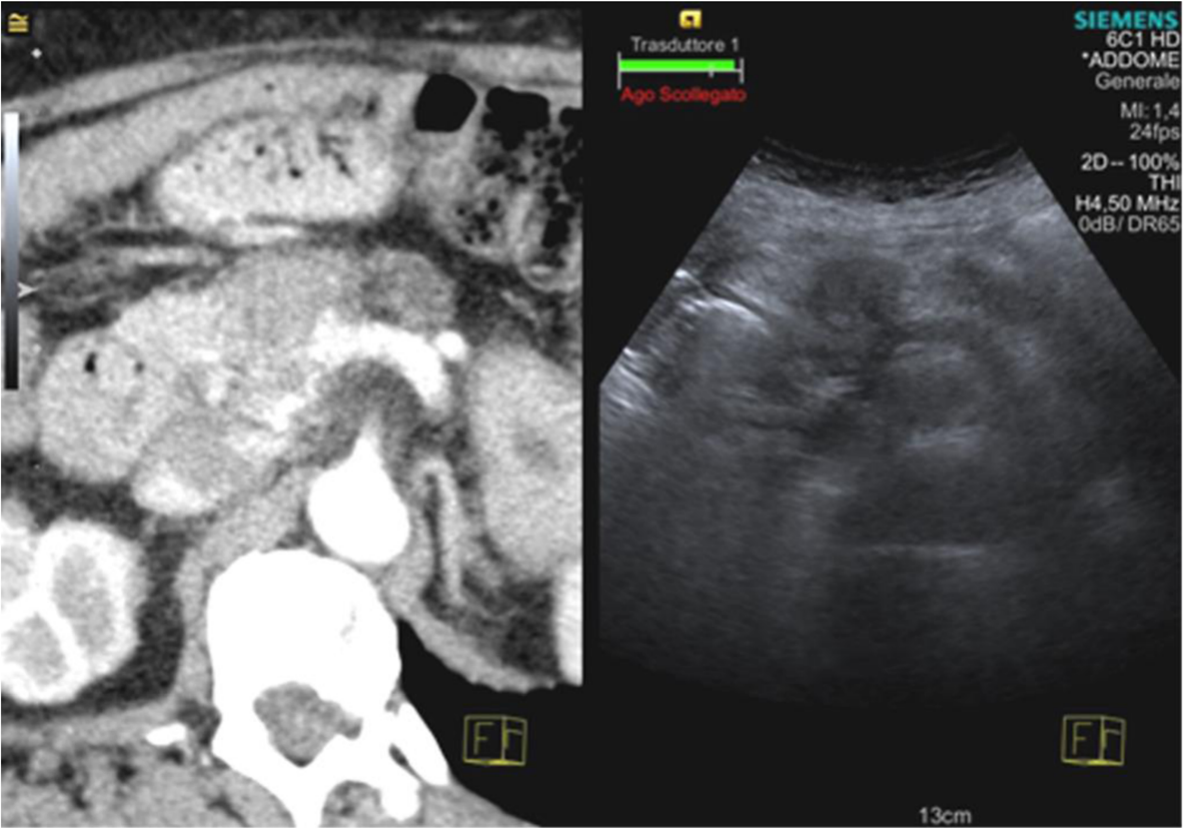
CT-US fusion imaging. Biopsy of pancreatic neck carcinoma, hypodense on CT and hypoechoic on the US

**Fig. 4:**
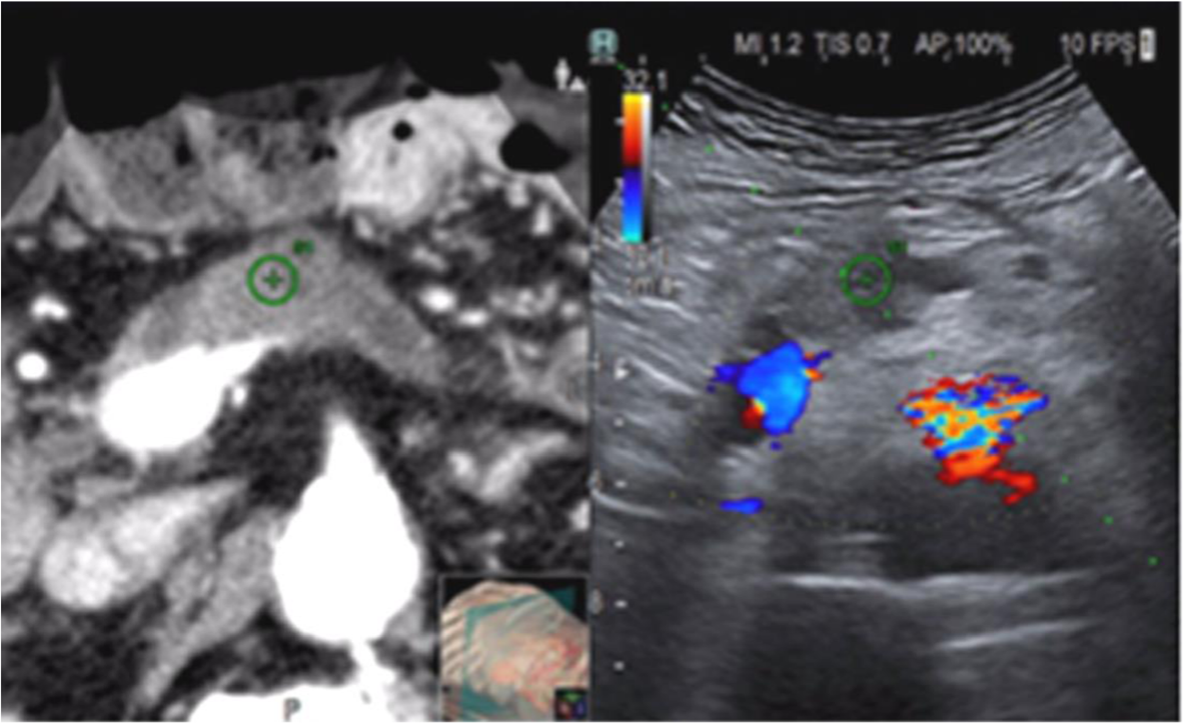
CT-US fusion imaging. Radiofrequency ablation planning of pancreatic neck carcinoma, hypodense on CT and hypoechoic on Doppler US (performed to highlight the peripancreatic vessels)

When image matching is complete, real-time US and CT/MR/PET images are arranged side-by-side or overlaid on the US monitor, displaying the same plane and moving synchronously together (Figs. 1, 2, 3 and 4). Thus, fusion imaging helps in the detection and localization of lesions with low conspicuity on standard B-mode US [4]. It is also possible to indicate the desired needle route, which can then be followed easily during the procedure.

There are many applications of fusion imaging, but given the relative novelty of the technology, most are still under investigation and require additional clinical trials. The current principal use of US fusion imaging is during hepatic interventional procedures. However, new applications in both intra- and extra-abdominal areas are emerging more and more.

## Liver

US is the method of choice for the interventional percutaneous approach to hepatic lesions. Its advantages in this area are manifold, both for diagnostic procedures such as biopsies and therapeutic procedures such as ablations. Firstly, it is a real-time method, allowing the liver to be constantly followed during respiratory movements. It allows identification of the most appropriate plane for needle insertion; this does not have to be an axial plane but can be oriented at will according to the circumstances. Furthermore, the use of color Doppler US allows us to identify major vascular structures (e.g., hepatic arteries, portal vein branches, hepatic veins) which should preferably be avoided during needle insertion. Finally, fused CEUS can be useful to increase the conspicuity of lesions to be treated and vascular structures to be avoided [3].

However, US has some weaknesses which can affect the success of the procedure. Firstly, it is an operator-dependent method and has a lower contrast resolution than CT and MR. Air contained in the hollow organs or in the biliary tract (in the case of pneumobilia) may limit the available acoustic window. Even air in the lung parenchyma, bones, and calcification (e.g., gallstones) interferes with the US. Furthermore, lesions and their margins are not always clearly visible on the B-mode US, even after the administration of contrast medium, particularly in the inhomogeneous and cirrhotic livers. The most common causes of mistargeting during US-guided radiofrequency ablation (RFA) of hepatocellular carcinoma (HCC) are confusion with cirrhotic nodules, poor conspicuity of the target lesion, and poor acoustic window [2]. Lesions located deep in the most distal sectors of the acoustic cone can be blurred and difficult to identify. Using fusion imaging technologies, it is possible to place beside or overlay upon the US image images from modalities that do not suffer from all these problems, such as CT and MR. In this way, radiologists can exploit all the strengths of the different methods in a single session, increasing the safety, speed, and results of the procedure and improving the confidence of the operator.

Some hepatic tumors which can be visualized by CT or MR cannot be seen on the US due to their small size, their location, or their echogenicity. In these cases, fusion imaging has been proven to enhance the conspicuity of HCC nodules and to increase the feasibility of percutaneous RFA of HCCs not visible on the conventional US [4–6]. If HCCs are still not visible after fusion imaging, anatomic landmarks surrounding the lesions can be used for correct needle placement [5]. Thus, with the use of fusion imaging, a larger population can benefit from US-guided ablation procedures instead of undergoing CT-guided ablation or major surgery, which are more harmful and expensive techniques.

Fusion imaging can also reduce false-positive lesion detection during US-guided RFA and consistently improve the detection of HCCs, especially when these are smaller than 2 cm [7]. The ability of fusion imaging to reduce false positives also applies to the evaluation of local tumor progression after RFA and TACE [8].

## Kidney

US is the usual first-line imaging method for the assessment of the kidneys. Thanks to their retroperitoneal location, in the lumbar region below the rib cage, an excellent acoustic window is generally available, without the interposition of air-containing structures or bones. Most renal lesions are incidental findings and are frequently asymptomatic. The main utility of US is the precise discrimination of solid lesions from cystic lesions. However, with only B-mode US, it can often be difficult to distinguish between simple and complex or neoplastic cysts. Even in the case of solid renal lesions, there may be difficulties with the B-mode US in terms of detection and characterization. For these reasons, it is often necessary to exploit second-level methods such as CT and MR for the study of renal lesions. CEUS is also emerging as a useful technique to study cystic lesions and their related septal vascularization, as well as solid lesions, during ablative procedures [9, 10]. In interventional procedures, fusion imaging can be extremely helpful in identifying the renal lesions to be treated, especially those with poor conspicuity on normal B-mode US. The combination of CEUS with fusion imaging is effective in the classification of indeterminate renal lesions and can also improve the characterization of cystic lesions [11]. Therefore, in the case of tumors, the use of CEUS with fusion imaging allows minimization of the risk of treating benign lesions surgically or the risk of missing cancer [12].

When targeting renal lesions during a percutaneous procedure, fusion imaging can help in recognizing the most appropriate part of the lesion to biopsy (especially for cystic lesions) or the best position to place the electrodes for ablation. In the case of multiple lesions, fusion imaging allows us to distinguish the specific lesion to be treated with greater confidence, allowing the margins of the lesion to be more precisely distinguished. Furthermore, the use of fusion imaging is valuable in determining the correct path for the needle, avoiding harm to structures such as the renal vessels, renal pelvis, adrenal glands, spleen, and colon.

However, the use of fusion imaging in renal disease is still under investigation in the literature and requires further clinical studies.

## Pancreas

When used by experienced operators, US allows the pancreas to be studied in excellent detail. As US is a real-time method, radiation-free, and can be performed at the bedside, it is an important aid for guiding pancreatic percutaneous procedures such as ablation of lesions or drain positioning [13]. However, since the pancreas is a retroperitoneal organ, it may be difficult to visualize it entirely if there is an interposition of hollow organs; this is particularly true with respect to the tail, sometimes called the “blind area” of the pancreas. CT and MR, on the other hand, are superior to US in permitting full visualization of the pancreas, providing clearer demonstration of its relationships to the delicate surrounding structures, lying behind the colon and stomach and in close contact with the duodenum, the portal vein and major arterial structures such as the aorta, the celiac axis, and the superior mesenteric artery. For this reason, fusion imaging can be an extremely useful tool to recognize and avoid damaging these structures during US-guided percutaneous procedures. It has been shown that performing drainage of pancreatic necrosis using US fusion imaging is superior to classic B-mode US in terms of safety, efficiency, and hospitalization length and costs [14]. US fusion imaging also allows better visualization of the “blind area” when it is not clearly shown with normal B-mode US [15].

Clinical indications for fusion imaging of the pancreas can therefore be summarized as guidance for biopsy and drainage and percutaneous treatment of pancreatic cancer such as radiofrequency ablation or irreversible electroporation.

Even in these circumstances, the use of fusion imaging in pancreatic disease is not well-described in the literature and requires further clinical studies to confirm its validity.

## Limitations of fusion imaging

One of the most challenging limitations affecting US fusion imaging is the risk of mistargeting a lesion. In hepatic ablation, mistargeting normally occurs in about 2% of cases and is principally due to the small size of the lesion or confusion with the surrounding pseudo lesions such as regenerative nodules. Lesions located in subcapsular or subphrenic areas, as well as lesions with poor conspicuity, can also be missed [9].

Another limitation of US fusion imaging is the need to synchronize a static image from a CT or MR study with the breathing motion and changing position of the patient, in particular when approaching a subdiaphragmatic organ such as the liver [10]. During the breathing cycle, the movement of the liver is complex and includes translations and rotations. During breathing, the peripheral regions of the liver move more widely than the central ones, which are more fixed in position by the presence of the hepatic pedicle. In the periphery of the liver, there are also fewer anatomical landmarks such as the vessels. For this reason, registration error during fusion imaging especially affects the peripheral portions of the liver and patients with large respiratory movements [11]. Although retroperitoneal, registration errors may also affect the kidney, as it is a mobile organ subject to movement with breathing. Registration errors occur when the respiratory phase of the reference examination is different from that during image synchronization. Therefore, MR, which is normally performed in expiration (as opposed to CT, usually performed during inspiration), is more comparable with the patient’s breathing status during the interventional procedure and is therefore less associated with registration errors [12].

The pancreas is a less mobile organ during breathing and is therefore theoretically less affected by registration errors. However, visualization of the pancreas often requires that pressure be applied with the probe on the upper abdomen to displace the overlying hollow organs. This can change the relationships between abdominal structures, which can affect the matching between the US and CT/MR images.

Finally, while fusion imaging is increasingly proving to be a promising technology, further randomized clinical trials are needed to define its presumed superiority over cognitive fusion during image-guided procedures.

## Conclusion

US fusion imaging is a relatively novel technique in the abdominal US panorama. Its ability to exploit all the strengths of multiple imaging methods used together in real time makes it a tool of great value when performing a percutaneous procedure. Increasing the confidence of the operator, it allows better visualization of the abdominal structures and more precise planning of needle paths, avoiding delicate structures, minimizing radiation exposure, and so increasing safety and efficiency (and decreasing cost) of these procedures.
